# Targeting Dendritic Cells in Cancer Immunotherapy: From Immune Priming to Tumor Microenvironment Reprogramming

**DOI:** 10.3390/ijms27219607

**Published:** 2026-10-27

**Authors:** Serena Zanotta, Domenico Galati

**Affiliations:** Hematology-Oncology and Stem-Cell Transplantation Unit, Department of Onco-Hematology and Innovative Diagnostics, Istituto Nazionale Tumori—IRCCS—Fondazione G. Pascale, IRCCS, 80131 Napoli, Italy

**Keywords:** dendritic cells, cancer immunotherapy, antigen presentation, cDC1, immune checkpoint blockade, tumor microenvironment, DC vaccines, FLT3L, neoantigen vaccine, mRNA vaccine

## Abstract

Dendritic cells (DCs) are specialized antigen-presenting cells that shape antitumor immunity by integrating antigen uptake, processing, presentation, co-stimulation, and cytokine-mediated T-cell instruction. Their function is therefore critical for generating tumor-specific T-cell responses and may influence responsiveness to immune checkpoint inhibitors. Although early ex vivo DC vaccines established feasibility, safety, and immunogenicity, clinical efficacy has been heterogeneous. Sipuleucel-T demonstrated an overall-survival benefit in metastatic castration-resistant prostate cancer, whereas DCVax-L produced encouraging but methodologically debated survival signals in glioblastoma. More recently, individualized neoantigen mRNA vaccination combined with pembrolizumab has shown durable recurrence-free survival benefit in resected high-risk melanoma. This review examines the biological rationale for DC-directed immunotherapy, tumor-induced DC dysfunction, next-generation DC platforms, in vivo DC targeting, DC-derived extracellular vesicles, negative and controversial clinical evidence, and rational combinations with checkpoint blockade and tumor-microenvironment-modulating strategies. The field is evolving from the use of DCs as cellular vaccine vehicles toward programmable immune-priming approaches that integrate antigen quality, DC functional state, spatial organization, and tumor microenvironment features.

## 1. Introduction

Dendritic cells (DCs) are professional antigen-presenting cells that play a central role in the initiation and regulation of antitumor immunity. Since their original identification by Steinman and Cohn, DCs have been recognized as immune sentinels able to acquire antigens, process them, migrate to secondary lymphoid tissues, and activate adaptive immune responses through peptide presentation by major histocompatibility complex (MHC) molecules and co-stimulatory signaling [1,2]. In cancer, these functions are essential because tumor-specific cytotoxic T-cell responses require not only tumor antigen availability, but also efficient antigen presentation within an inflammatory and co-stimulatory context capable of supporting T-cell priming, expansion, trafficking, and effector differentiation [2,3].

The clinical success of immune checkpoint inhibitors (ICIs) has demonstrated the therapeutic relevance of restoring antitumor T-cell function. However, checkpoint blockade is most effective when tumor-specific T cells are already present or can be generated during therapy. Reduced intratumoral recruitment of dendritic cells, particularly Conventional type 1 DCs (cDC1s), and defective DC-dependent T-cell priming can impair effector T-cell infiltration and contribute to resistance to programmed cell death protein 1 (PD-1)/programmed death-ligand 1 (PD-L1) blockade [4,5,6]. This places DC function upstream of checkpoint-regulated T-cell responses and helps determine whether effective antitumor immunity can be initiated and sustained.

The therapeutic use of DCs has been investigated for more than two decades. Ex vivo-generated DC vaccines have been evaluated in both solid and hematologic malignancies, most commonly using autologous monocyte-derived DCs loaded with tumor-associated antigens, tumor lysates, tumor ribonucleic acid (RNA), or defined peptide antigens [7,8]. Sipuleucel-T, an autologous cellular immunotherapy targeting prostatic acid phosphatase, remains the best-established example. In the pivotal IMPACT trial, sipuleucel-T improved median overall survival from 21.7 to 25.8 months compared with placebo, corresponding to an absolute survival gain of 4.1 months and a 22% relative reduction in the risk of death (hazard ratio [HR] 0.78; 95% confidence interval [CI], 0.61–0.98; *p* = 0.03) [9]. Importantly, this survival benefit was not accompanied by a clear improvement in time to objective disease progression, an endpoint discordance discussed further in Section 4.5 [9].

In glioblastoma, DCVax-L has shown encouraging survival results, although their interpretation remains methodologically challenging. In the phase 3 study, median overall survival was 19.3 versus 16.5 months in newly diagnosed glioblastoma (HR 0.80; *p* = 0.002) and 13.2 versus 7.8 months in recurrent disease (HR 0.58; *p* < 0.001) compared with matched external controls [10]. However, the use of external rather than concurrent randomized controls, together with crossover and potential differences between study populations, limits the certainty with which the observed survival benefit can be attributed to DCVax-L [11].

Together, these studies capture the mixed clinical picture of DC-based immunotherapy. DC vaccines are generally well tolerated and can induce measurable tumor-specific immune responses, but these effects do not consistently translate into improved clinical outcomes. Recent trials have shown substantial heterogeneity, ranging from enhanced antigen-specific T-cell responses without significant improvement in clinical endpoints to encouraging but still preliminary signals of activity [12,13,14]. This variability likely reflects differences in DC subset and functional state, antigen selection, treatment setting, and the immunosuppressive tumor microenvironment (TME) [2].

The field is therefore moving beyond the traditional use of DCs as ex vivo vaccine vehicles toward broader strategies that modulate DC differentiation, activation, antigen presentation, and interactions within the TME. Current approaches include optimized ex vivo DC products, Fms-like tyrosine kinase 3 ligand (FLT3L)-based expansion or differentiation, receptor-mediated antigen delivery, CD40, Toll-like receptor (TLR) and stimulator of interferon genes (STING) agonists, nanoparticle and messenger RNA (mRNA)-based platforms, and DC-derived extracellular vesicles [2,15,16,17]. Increasingly, these strategies are being combined with checkpoint blockade and other treatments that modulate the tumor microenvironment [18,19].

This review examines DCs as therapeutic targets in cancer immunotherapy, focusing on the biological and translational principles that guide their therapeutic exploitation. Particular emphasis is placed on clinical evidence, negative and controversial studies, single-cell and spatial profiling of tumor-infiltrating DCs, and the development of biomarker-driven combination strategies.

## 2. Biological Rationale for Targeting Dendritic Cells in Cancer

The rationale for targeting DCs in cancer derives from their ability to link tumor antigen recognition with adaptive immune activation. Among antigen-presenting cells, DCs are particularly efficient at priming naïve T cells through coordinated antigen presentation, co-stimulatory signaling, and cytokine-mediated instruction [2,3]. In tumors, these functions support the generation of cytotoxic CD8+ T-cell responses, CD4+ T-cell help, and durable antitumor immune memory [2,20].

cDC1s are particularly relevant to antitumor immunity. These cells are specialized in cross-presenting exogenous tumor antigens through MHC class I molecules and are therefore critical for priming tumor-specific CD8+ T-cell responses [3,20,21]. Clinically, higher intratumoral cDC1 abundance and their spatial proximity to CD8+ T cells have been associated with T-cell infiltration and clinical benefit from checkpoint inhibitors across multiple tumor types [4,22]. This association supports strategies aimed at expanding cDC1s, enhancing their recruitment, or promoting their activation.

DC-mediated antitumor immunity is also shaped by reciprocal interactions with other innate immune populations. Tumor-infiltrating natural killer (NK) cells can promote cDC1 accumulation through CCL5 and XCL1 and can sustain intratumoral DC abundance through FLT3L, establishing an NK-DC axis that favors cross-priming and subsequent CD8+ T-cell responses [4,20,23]. Conversely, suppressive myeloid populations, including tumor-associated macrophages and myeloid-derived suppressor cells, can interfere with DC recruitment, maturation, and antigen-presenting function through cytokine- and metabolite-mediated mechanisms [2,20]. These interactions indicate that the activity of DC-directed platforms depends not only on the targeted DC compartment but also on how each intervention mobilizes or reprograms the surrounding innate immune network.

Conventional type 2 DCs (cDC2s) contribute to antitumor immunity primarily by presenting tumor antigens to CD4+ T cells and promoting helper T-cell differentiation [20,24]. CD4+ T-cell help can in turn support the magnitude and persistence of cytotoxic responses through DC licensing, cytokine production, and memory formation. Although cDC1s are central to cross-presentation, effective antitumor immunity depends on coordinated interactions among multiple DC subsets and other immune and stromal components within the TME [2,3].

Plasmacytoid DCs (pDCs) add further complexity to antitumor immunity. When appropriately activated, their production of type I interferons can support antitumor immune responses. Within the tumor microenvironment, however, pDC function may be suppressed or reprogrammed toward a tolerogenic phenotype, contributing to impaired effector responses and immune escape [25,26]. Thus, the role of pDCs is highly context-dependent and reflects their activation state and the surrounding microenvironment.

From a therapeutic perspective, this functional heterogeneity has direct implications for platform selection. Strategies designed to enhance cross-presentation or CD8+ T-cell priming are most naturally aligned with cDC1 biology, whereas approaches intended to strengthen CD4+ T-cell help may depend more heavily on cDC2 engagement. pDC-directed strategies are less straightforward because type I interferon production can be immunostimulatory, while tumor-conditioned pDCs may acquire tolerogenic functions. Thus, the efficacy of a DC-based intervention is likely to depend on which subset is targeted or expanded, its activation state, and whether the surrounding TME preserves the functional program that the platform is intended to exploit [2,20,24,25,26].

Monocyte-derived DCs (moDCs) have historically been the most widely used cells for ex vivo DC vaccine manufacturing because they can be readily generated from peripheral blood monocytes using GM-CSF and IL-4 [27,28]. However, moDCs are phenotypically and functionally distinct from naturally occurring conventional DC subsets, and their immunogenic properties are strongly influenced by differentiation and maturation conditions and antigen-loading strategies [2,28]. For vaccine development, antigen loading alone is insufficient: DC maturation, migratory capacity, co-stimulatory profile, cytokine production, and resistance to tumor-induced suppression also shape efficacy [2,28].

Recent single-cell and spatial profiling studies have expanded the conventional subset-based view of tumor-infiltrating DCs, revealing distinct developmental, migratory, inflammatory, and regulatory states shaped by the tumor microenvironment [29,30]. Pan-cancer single-cell analyses have further defined DC heterogeneity across malignancies, including rare subsets and tumor-associated transcriptional programs [29]. In metastatic melanoma, analysis of approximately 189,000 cells from 36 samples identified mature regulatory/migratory DC states associated with immune infiltration and progression-free survival following ICI-based therapy, with validation in an independent cohort of 318 patients [30]. Spatial profiling further indicates that the localization of DCs relative to T cells, stromal regions, tumor nests, and tertiary lymphoid-like structures may be as important as subset abundance alone [31]. A lineage-only view is therefore insufficient; DC-directed therapies should also account for functional state, spatial organization, and cellular interactions within the TME.

The relevance of DC biology extends beyond cellular vaccines. Receptor-mediated antigen delivery to DC-associated molecules, including C-type lectin domain containing 9A (CLEC9A)/dendritic cell natural killer lectin group receptor-1 (DNGR-1), DEC205/CD205, and X-C motif chemokine receptor 1 (XCR1)-associated pathways, exploits the specialized antigen-uptake and cross-presentation capacity of selected DC subsets [16,32]. Similarly, CD40, TLR, and STING agonists can enhance DC activation, inflammatory cytokine production, and T-cell priming [19,33,34]. These approaches may be particularly relevant in poorly inflamed tumors, where insufficient DC activation and T-cell priming can limit responsiveness to checkpoint blockade [19,34].

DC vaccination and checkpoint blockade have distinct functional roles rather than acting at strictly separated stages of the antitumor response. DC-directed vaccination promotes antigen-specific priming and expansion of tumor-reactive T cells, whereas ICIs provide antigen-nonspecific modulation of inhibitory checkpoint signaling, thereby restoring or sustaining the activity of T cells that have already encountered cognate antigen. These mechanisms are complementary and support the combined use of DC-targeted approaches, checkpoint blockade, and TME-modulating therapies [3,18,19].

## 3. Tumor-Driven Dendritic Cell Dysfunction and Immune Escape

Tumor-induced impairment of DC differentiation, maturation, migration, and antigen-presenting function is an important mechanism of immune escape. Within the TME, suppressive factors such as vascular endothelial growth factor (VEGF), interleukin-10 (IL-10), transforming growth factor beta (TGF-β), prostaglandin E2 (PGE2), adenosine, lactate, and hypoxia-associated signals can impair DC maturation, migration, cytokine production, and antigen presentation [2,35,36,37]. These changes can shift DCs from immunogenic toward dysfunctional or tolerogenic states, limiting effective T-cell priming and promoting tumor immune escape [2,36,37]. A schematic overview of antitumor DC function and tumor-driven dysfunction is shown in Figure 1.

Mechanistically, these abnormalities can be separated into two broad, although overlapping, categories. Some directly compromise antigen presentation by reducing antigen processing or cross-presentation, MHC/co-stimulatory function, or cytokine support, whereas others primarily limit the number or state of competent DCs by impairing recruitment, differentiation, maturation, or migration within the TME [2,4,5,6,35,36,37]. This distinction has therapeutic implications: defects in antigen presentation may favor strategies that improve antigen delivery or DC activation, whereas recruitment or maturation defects may require DC expansion, vascular normalization, or relief of suppressive TME signals [5,35,37].

DC dysfunction is not uniform across malignancies. In solid tumors, impaired recruitment or functional silencing of cDC1s may reduce CD8+ T-cell priming and limit response to checkpoint blockade [4,5,6]. In hematologic malignancies, malignant cells and disease-specific microenvironments can induce distinct defects in DC phenotype and antigen-presenting function. In multiple myeloma, DC subsets display impaired antigen processing and presentation and reduced capacity to support effective T-cell responses [38,39]. In acute myeloid leukemia, the limited immunogenicity of leukemic blasts has prompted strategies aimed at generating leukemia-derived DCs capable of presenting leukemia-associated antigens and activating antileukemic T-cell responses [40].

The therapeutic implications differ according to the immune context of each tumor. DC expansion or activation may be insufficient when the TME impairs DC maturation, excludes effector cells, or promotes tolerogenic reprogramming. Conversely, efficacy may be enhanced by combining DC-directed approaches with therapies that induce immunogenic tumor cell death, relieve immune exclusion, normalize tumor vasculature, or counteract suppressive cytokine pathways [35,37,41,42].

Tumor antigenicity alone is not sufficient to ensure effective antitumor immunity. Even tumors with a high mutational burden or abundant neoantigens may remain poorly responsive if DCs fail to efficiently cross-present tumor antigens and prime CD8+ T-cell responses [18]. Restoring DC function may therefore be particularly important in immune-excluded or poorly inflamed tumors, where DC-directed strategies may act as immune initiators and convert non-inflamed lesions into tumors more susceptible to checkpoint blockade [6].

## 4. Therapeutic Strategies to Restore or Exploit Dendritic Cell Function

Therapeutic strategies targeting DCs can be grouped into four major platforms: classical ex vivo-generated DC vaccines, next-generation DC products designed to better reproduce physiological DC biology, in vivo DC-targeting approaches, and cell-free strategies based on DC-derived extracellular vesicles [2,16,17]. Although these approaches differ in biological rationale, manufacturing complexity, and clinical maturity, they share the goal of improving tumor antigen presentation and promoting effective antitumor T-cell responses. Negative and controversial clinical studies further help define the biological and therapeutic limitations of each platform. These therapeutic platforms and their translational roadmap are summarized in Figure 2.

### 4.1. Classical Ex Vivo DC Vaccines: Lessons Learned

Classical DC vaccines are based on ex vivo generation of autologous DCs, most commonly from peripheral blood monocytes cultured with GM-CSF and IL-4, followed by antigen loading, maturation, and reinfusion. Antigen-loading strategies have included defined tumor-associated peptides, whole tumor lysates, apoptotic tumor cells, tumor RNA, and mRNA encoding tumor antigens, while personalized neoantigen vaccination has broadened antigen-specific strategies in cancer immunotherapy [7,8,43]. The therapeutic rationale is that antigen-loaded DCs should migrate to lymphoid tissues, present tumor-derived peptides through MHC molecules, and activate tumor-specific T cells.

Clinical studies indicate that DC vaccines are generally feasible and well tolerated, although their clinical efficacy remains heterogeneous [12,13,14]. Sipuleucel-T demonstrated an overall survival benefit in metastatic castration-resistant prostate cancer, with median overall survival of 25.8 versus 21.7 months and an HR for death of 0.78 [9]. DCVax-L reported median overall survival of 19.3 versus 16.5 months in newly diagnosed glioblastoma and 13.2 versus 7.8 months in recurrent disease compared with matched external controls [10]. By contrast, other trials have demonstrated vaccine-induced tumor-specific immune responses without consistent improvement in clinical endpoints, reinforcing the recurrent disconnect between immunogenicity and therapeutic benefit.

Several biological and technical factors may explain the gap between vaccine immunogenicity and clinical efficacy. Monocyte-derived DCs are practical to manufacture but do not fully reproduce the functional specialization of naturally occurring cDC1 and cDC2 subsets [2,27]. DC maturation and migratory competence are also critical, as they determine co-stimulatory capacity, cytokine production, lymph-node trafficking, and effective T-cell priming [28,44]. Antigen selection represents another major determinant: tumor-associated antigens may be limited by immune tolerance, whereas neoantigens offer greater tumor specificity but require individualized identification, prioritization, and manufacturing workflows [45]. Finally, vaccine-induced DCs must remain functional within an immunosuppressive TME characterized by inhibitory cytokines, suppressive myeloid populations, metabolic constraints, and checkpoint signaling [2,36].

Classical DC vaccination therefore shows that antigen loading and immunogenicity alone are poor predictors of therapeutic efficacy. Clinical activity depends on the overall functional quality of the DC product, including subset identity, maturation state, migratory capacity, cytokine profile, antigenic cargo, and interaction with the TME. These limitations have driven the development of engineered DC products, subset-specific strategies, and rational combination approaches.

### 4.2. Next-Generation DC Products: Subset Composition and Functional Programming

Next-generation DC platforms are designed to overcome the limitations of conventional monocyte-derived products by improving subset composition, activation state, antigen-processing capacity, and resistance to tumor-induced suppression. Because generation conditions directly shape DC function, the manufacturing strategy itself can influence therapeutic performance [2,27].

FLT3L-based systems are particularly relevant because FLT3L is a physiological regulator of DC development and supports the generation of conventional DC subsets. In preclinical models, FLT3L-containing cultures can generate cDC-like populations with efficient cross-presentation capacity [46]. More recently, FLT3L combined with GM-CSF was shown to generate DCs enriched in conventional DC subsets, with enhanced cross-presentation, stronger tumor-specific CD8+ T-cell responses, increased cytotoxic T-cell infiltration, and reduced immunosuppressive features within the TME [47]. For next-generation DC products, functional potency may therefore be more informative than phenotype alone. Relevant parameters include cross-presentation, cytokine production, CCR7-dependent migration, and T-cell stimulatory capacity. Viability, sterility, and basic immunophenotyping may therefore be insufficient to define the functional potency of a DC product [27,44].

Disease-specific DC programming represents another therapeutic strategy. In acute myeloid leukemia, leukemia-derived DCs (DCleu) can be generated from leukemic blasts, allowing presentation of a broad patient-specific leukemia-associated antigen repertoire without prior antigen selection [48,49]. These cells can stimulate leukemia-reactive T-cell responses and enhance antileukemic cytotoxicity ex vivo [48]. Importantly, the composition and concentration of immunomodulatory cocktails strongly influence DC/DCleu generation, immune activation, antileukemic activity, and off-target toxicity [40]. For DCleu, therapeutic potential therefore depends on controlled functional programming rather than cellular identity alone.

Next-generation DC products may also incorporate genetic or molecular engineering to enhance cytokine production, antigen presentation, or DC activation and persistence. Such approaches are conceptually attractive but require precise, cell-specific control, because manipulation of intracellular regulatory pathways can produce context-dependent or even detrimental effects, as illustrated by the impaired antitumor immunity observed after SHP-1 loss in CD11c+ cells [50].

### 4.3. In Vivo DC Targeting and Activation

In vivo DC targeting aims to deliver antigens or activating signals directly to endogenous DCs, thereby avoiding some limitations of ex vivo manufacturing. This approach exploits the physiological antigen-uptake, migratory, and cross-presenting functions of endogenous DC subsets [16,20].

One strategy is to expand DC populations before therapeutic activation. FLT3L can increase conventional DC abundance in peripheral and tumor-associated compartments, thereby increasing the availability of antigen-presenting cells for antitumor immune priming [5,51]. However, DC expansion alone is unlikely to be sufficient and generally requires concomitant antigen release and activating signals. For this reason, FLT3L-based strategies have been combined with radiotherapy, TLR agonism, and checkpoint blockade to promote DC recruitment, cross-presentation, and effective T-cell priming [52,53].

Receptor-mediated antigen delivery is another major in vivo strategy. Targeting antigens to receptors expressed by DC subsets, including DEC205, CLEC9A/DNGR-1, and other endocytic receptors, can increase antigen uptake and presentation efficiency [16,32]. CLEC9A is particularly relevant because it is selectively expressed on cDC1s and recognizes dead cell-associated material, thereby promoting cross-presentation and CD8+ T-cell priming [16,32]. However, antigen targeting in the absence of appropriate DC activation can favor tolerogenic rather than immunogenic responses. Concomitant adjuvant or inflammatory signals are therefore often required to achieve effective cytotoxic T-cell immunity [54].

Innate immune agonists provide a complementary mechanism for activating DCs in vivo. TLR agonists can promote DC maturation, cytokine production, co-stimulatory molecule expression, and T-cell activation [33]. STING agonists can induce type I interferon signaling and promote cDC1-dependent cross-priming of antitumor CD8+ T-cell responses [19]. CD40 agonists can further license DCs and enhance tumor-specific T-cell priming [34]. Despite this strong biological rationale, clinical translation remains challenging because effective intratumoral immune activation must be achieved while limiting systemic inflammation and other on-target toxicities [15,55].

Nanoparticle and mRNA-based platforms are increasingly used to enhance in vivo antigen delivery to dendritic cells and other antigen-presenting cells. Nanoparticle formulations can protect nucleic acids, co-deliver antigen and adjuvant signals, and facilitate delivery to lymphoid tissues, thereby promoting antigen uptake and expression by APCs [56,57,58]. mRNA vaccines, including individualized neoantigen vaccines, rely on endogenous APCs for antigen expression, presentation, and subsequent T-cell priming. Their activity therefore depends on both efficient delivery and the innate immune signals generated by the formulation [57]. mRNA vaccine platforms should therefore be considered endogenous APC-dependent immune-programming strategies rather than DC-specific interventions. DCs can make an important contribution to antigen uptake, presentation, and T-cell priming, but other antigen-presenting cell populations may also participate depending on the formulation and tissue distribution [56,57,58]. Accordingly, clinical activity of mRNA vaccination should not be interpreted as direct evidence of DC-specific targeting, but rather as evidence for a broader antigen-presenting-cell network in which DCs are often important contributors.

### 4.4. DC-Derived Exosomes and Cell-Free Approaches

DC-derived exosomes, or dexosomes, represent a cell-free extension of DC-based immunotherapy. These extracellular vesicles can carry MHC-peptide complexes, co-stimulatory molecules, adhesion molecules, heat shock proteins, nucleic acids, and immunologically active cargo capable of modulating T cells, NK cells, and other immune populations [17,59]. Their therapeutic rationale is based on the possibility of preserving selected immunostimulatory functions of DCs while reducing limitations associated with living cell products, including variability in migration, viability, and post-infusion behavior [59].

Preclinical studies have demonstrated the antitumor potential of DC-derived exosomes, while early-phase clinical trials in melanoma and non-small cell lung cancer established their feasibility and immunological activity [60,61]. However, clinical development remains limited. Major challenges include vesicle heterogeneity, manufacturing standardization, potency assessment, biodistribution, and reproducible antigen loading [62]. More recently, incorporating immunogenic cell death signals into dexosome production has been proposed as a strategy to enhance their antigenic and adjuvant properties [17].

Dexosomes are biologically distinct from living DCs and cannot dynamically adapt to the tumor microenvironment. Their therapeutic activity therefore depends on the composition of their cargo, surface molecules, and delivery, making them attractive candidates for engineered and combination immunotherapy approaches.

### 4.5. Negative and Methodologically Debated Clinical Evidence

A critical appraisal of DC-based immunotherapy requires consideration of negative and methodologically challenging trials. Immunological activity does not necessarily translate into clinical benefit, and antigen-specific T-cell responses may occur without improvement in survival outcomes.

The randomized phase 3 MIND-DC trial provides a clear example. In this study, “natural DC” (nDC) vaccination referred to an autologous product composed of naturally circulating CD1c+ conventional DCs and plasmacytoid DCs, rather than monocyte-derived DCs generated ex vivo. These DC subsets were isolated from peripheral blood, loaded with tumor-associated antigens, and administered intranodally as adjuvant therapy after complete resection of stage IIIB/C melanoma. A total of 148 patients were randomized to nDC vaccination or placebo. Although nDC vaccination was well tolerated and induced functional antigen-specific T-cell responses, it did not improve recurrence-free survival (RFS): the 2-year RFS rate was 36.8% with nDC vaccination versus 46.9% with placebo, and median RFS was 12.7 versus 19.9 months, respectively (HR 1.25; 90% CI, 0.88–1.79; *p* = 0.29) [12]. MIND-DC therefore illustrates a central limitation of the field: robust vaccine-induced immune responses do not necessarily ensure clinical efficacy, and the biological and disease-specific determinants of that disconnect remain critical to define.

DCVax-L showed encouraging overall survival results in both newly diagnosed and recurrent glioblastoma [10]. However, interpretation remains limited by the use of external rather than concurrent randomized controls, the crossover design, and methodological changes introduced during the prolonged study period, which complicate attribution of the observed survival benefit specifically to the vaccine [11,63]. A different issue emerges from the IMPACT trial of sipuleucel-T, in which an overall survival benefit was observed without a corresponding improvement in time to objective disease progression [9]. This discordance may reflect the delayed kinetics of immune-mediated effects and the limitations of conventional progression-based endpoints for some immunotherapeutic strategies. Future DC-directed trials should therefore integrate clinically meaningful endpoints with immune monitoring and biomarker-based patient stratification.

Negative and controversial studies point to several recurrent barriers to DC-based immunotherapy, including insufficient tumor antigenicity, impaired DC recruitment, maturation or migration, suboptimal T-cell priming and effector function, immune exclusion, and metabolic suppression within the tumor microenvironment [2,18,36]. Recognizing these biological limitations is essential for the design of more rigorous, biomarker-driven, and mechanistically informed clinical trials.

## 5. Combination Strategies with Immune Checkpoint Blockade and Other Immunomodulatory Approaches

The combination of DC-targeted therapies with immune checkpoint inhibitors (ICIs) is supported by a strong mechanistic rationale. Checkpoint blockade can restore or sustain antitumor T-cell activity but cannot fully compensate for insufficient tumor antigen presentation and T-cell priming. Conversely, DC activation can initiate and amplify tumor-specific T-cell responses, which may subsequently be restrained by inhibitory checkpoint pathways within the TME. Combining DC-directed interventions with ICIs therefore targets complementary stages of antitumor immunity by enhancing T-cell priming while relieving inhibitory regulation [2,64].

This broader innate context is relevant across therapeutic platforms. FLT3L-based approaches, TLR/STING agonists, cytokine-based regimens, and TME-modulating treatments may influence NK-cell recruitment or the balance between stimulatory and suppressive myeloid populations, thereby indirectly altering DC abundance and function in addition to their direct effects on DCs [2,4,15,19,23].

Several therapeutic scenarios support this approach. In poorly inflamed tumors, DC-targeted interventions can enhance antigen presentation and T-cell priming. FLT3L-mediated DC expansion, CD40 activation, STING or TLR agonism, radiotherapy, and immunogenic chemotherapy can provide complementary signals by increasing DC availability, antigen release, innate immune activation, and cross-presentation [18,19,42,52]. Once tumor-specific T-cell responses are established, PD-1/PD-L1 blockade can further support their antitumor activity [65]. In immune-excluded tumors, TGF-β inhibition or vascular normalization may facilitate immune-cell trafficking and T-cell infiltration [41,66]. DC activation may also broaden the repertoire of tumor-reactive CD8+ T-cell responses, including responses against otherwise weakly immunogenic neoantigens [18].

Beyond checkpoint blockade, DC vaccination can also be combined with innate immune agonists and cytokine-based interventions. TLR agonists can reinforce DC maturation and inflammatory polarization and thereby improve the quality of vaccine-induced T-cell priming. In a randomized phase II study in malignant glioma, the addition of poly-ICLC (TLR3 agonist) or resiquimod (TLR7/8 agonist) to autologous tumor lysate-pulsed DC vaccination was safe and enhanced systemic interferon-related immune responses, with poly-ICLC producing the strongest interferon-associated signature [67]. Cytokine-based strategies may act at complementary levels: FLT3L and GM-CSF can increase DC availability or influence DC differentiation [5,47], whereas cytokines such as IL-2 have been explored to support downstream effector-cell expansion. Clinical results have been variable; in a phase I/II study of metastatic melanoma, adding low-dose IL-2 to mature DC vaccination did not produce a clear survival advantage [68]. These findings support a broader combination framework in which DC vaccination is paired not only with checkpoint blockade but also with innate adjuvants and cytokine-mediated immune conditioning, selected according to the biological deficit that limits antitumor immunity.

A particularly important contemporary example is individualized neoantigen mRNA vaccination combined with PD-1 blockade. In the randomized phase 2b KEYNOTE-942 trial, mRNA-4157/V940 (now intismeran autogene), an individualized mRNA-based neoantigen therapy, combined with pembrolizumab prolonged recurrence-free survival compared with pembrolizumab alone in patients with completely resected high-risk melanoma (HR for recurrence or death, 0.561; 95% CI, 0.309–1.017) [69]. At the planned 5-year follow-up, the benefit remained durable, with a 49% reduction in the risk of recurrence or death (HR 0.510; 95% CI, 0.294–0.887) and a 59% reduction in the risk of distant metastasis or death (HR 0.411; 95% CI, 0.200–0.843) compared with pembrolizumab alone [70].

Although individualized mRNA neoantigen vaccines are not classical ex vivo DC vaccines, they rely on endogenous antigen-presenting cells for antigen expression, presentation, and T-cell priming. Recent clinical studies have shown that these vaccines can induce robust neoantigen-specific T-cell responses when combined with checkpoint blockade [69,71]. Direct combinations of ex vivo DC vaccination with ICIs have also entered clinical testing, with heterogeneous results across tumor types [14,72]. In melanoma, DC vaccination combined with CTLA-4 blockade has induced broad and multifunctional tumor-specific T-cell responses in a subset of patients [73]. In glioblastoma, evidence for combining DC vaccination with PD-1 blockade remains preliminary and is currently supported mainly by case-level observations and ongoing clinical investigation [74]. In hematologic malignancies, DC-based vaccination has been explored in post-transplant and maintenance settings. In multiple myeloma, a randomized phase II study evaluated a DC/myeloma fusion vaccine combined with lenalidomide maintenance after autologous transplantation [13], whereas post-allogeneic transplantation DC/AML fusion vaccination has so far been reported from an early-phase study [75]. Multiple myeloma further illustrates how DC dysfunction, T-cell exhaustion, stromal-mediated immunosuppression, and antigenic complexity may collectively limit vaccine efficacy and provide a rationale for combination strategies [38].

Radiotherapy and selected chemotherapies can promote DC-dependent antitumor immunity by inducing immunogenic tumor cell death, increasing antigen release, and promoting the exposure or release of damage-associated molecular patterns that facilitate DC activation and antigen presentation [42,76]. These mechanisms can convert tumor cell death into an endogenous vaccination event, provided that DCs remain functionally competent. However, the immunological effects of cytotoxic therapy are highly context-dependent. Radiation dose, fractionation, treatment schedule, and the local tumor microenvironment can determine whether radiotherapy promotes or restrains DC maturation, migration, cross-priming, and subsequent antitumor T-cell responses [37,77,78].

Anti-angiogenic therapy is another relevant partner for DC-directed strategies. VEGF pathway inhibition can promote vascular normalization and enhance intratumoral infiltration of mature DCs and effector T cells [35,79]. Metabolic factors within the TME can also impair DC function. In particular, tumor-derived lactate can drive tolerogenic DC reprogramming and suppress antigen cross-presentation, whereas targeting the CD73–adenosine pathway can alleviate immunosuppression and enhance DC and T-cell activity [36,80].

Combination design should start from the dominant immune barrier rather than from the indiscriminate addition of immunomodulatory agents. Limited tumor antigenicity may favor neoantigen-based vaccination, whereas defective DC recruitment or activation may be addressed through DC-targeted strategies or innate immune agonists [18,19]. Checkpoint blockade may be particularly relevant when inhibitory T-cell signaling predominates [69] while stromal exclusion or metabolic suppression may require TME-directed interventions [36,41]. In this framework, tumor immune context and mechanistic biomarkers provide the basis for selecting DC-based combinations.

## 6. Comparative Overview of DC-Directed Platforms

Table 1 summarizes the principal DC-directed or DC-dependent therapeutic platforms discussed in this review. The purpose of the table is not to rank these approaches, but to clarify their mechanism, clinical maturity, representative evidence, and main limitations.

## 7. Conclusions: A Roadmap for DC-Targeted Immunotherapy

Future development of DC-targeted immunotherapy should focus on five priorities. First, therapeutic strategies should be matched to the dominant immune barrier, including limited antigenicity, defective DC recruitment or activation, immune exclusion, and metabolic suppression [18,19,36]. Second, clinical trials should prioritize meaningful clinical endpoints rather than rely primarily on immunogenicity, as immune responses do not necessarily translate into improved clinical outcomes [12]. Third, DC subsets, functional states, and spatial organization should be characterized using single-cell, spatial, and functional profiling [29,30,31]. Fourth, combination strategies should be mechanistically guided, as illustrated by the clinical benefit of individualized mRNA neoantigen vaccination combined with PD-1 blockade [69]. Beyond immune checkpoint blockade, combinations with innate immune agonists, including TLR agonists, and selected cytokine-based strategies may further enhance DC activation, antigen presentation, and T-cell priming, providing additional avenues for rational multimodal immunotherapy [33,67,68]. Future studies should therefore evaluate DC responses together with the mobilization and functional remodeling of other innate immune compartments, particularly NK cells and suppressive myeloid populations, as potential determinants of therapeutic efficacy [2,4,23]. Finally, negative trials should be systematically reported, as they help define the biological and clinical settings in which DC activation alone is insufficient [12].

DC-targeted immunotherapy is best viewed as a programmable immune-priming strategy rather than a single vaccine modality. Its success will depend on integrating antigen quality, DC functional state, tumor microenvironment features, and checkpoint dependence into biomarker-informed clinical trial design.

## Figures and Tables

**Figure 1:**
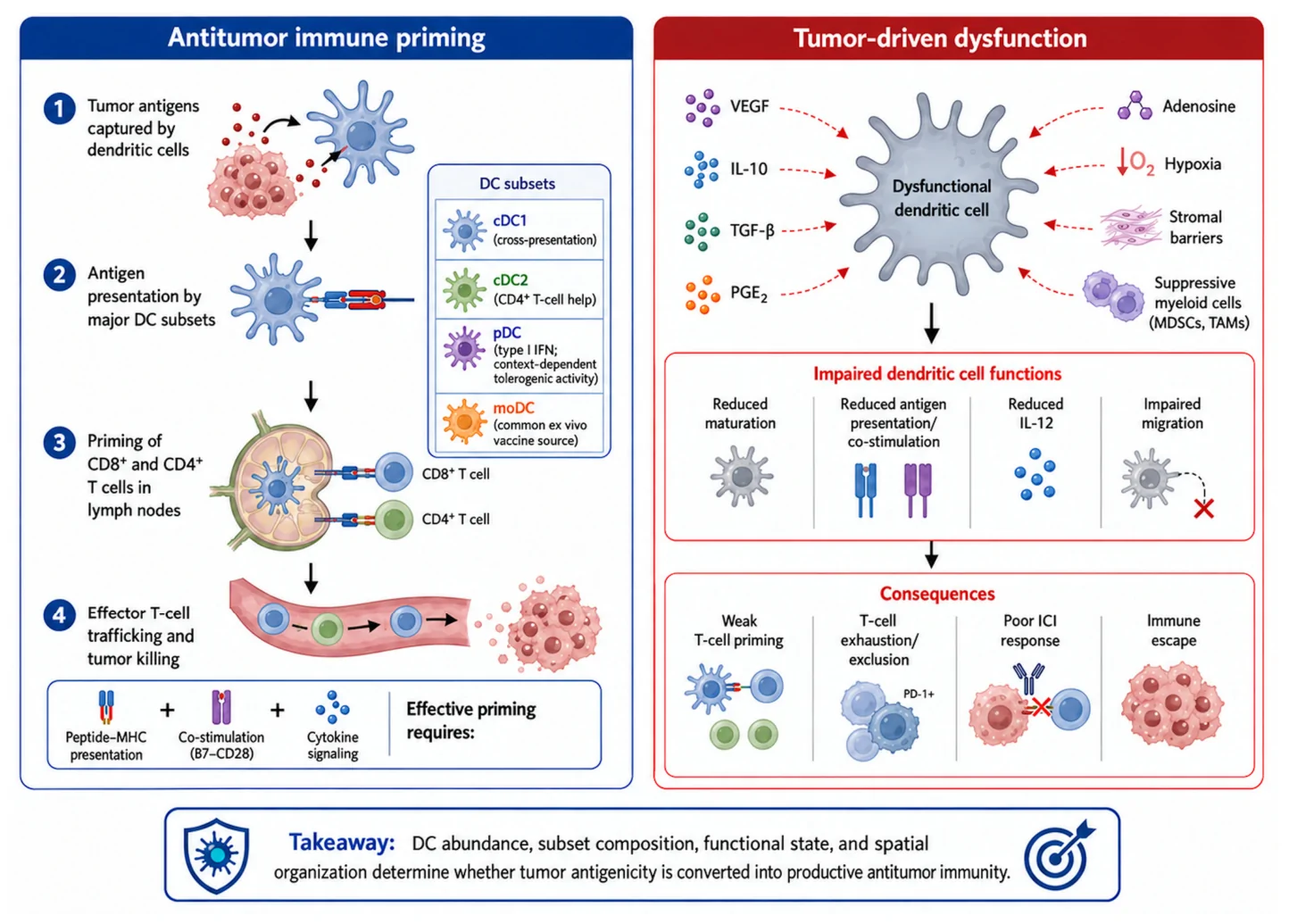
Dendritic cells in antitumor immunity and tumor-driven dysfunction. The left panel illustrates the role of dendritic cells (DCs) in antitumor immune priming. Tumor antigens are captured, processed, and presented by major DC subsets, leading to CD8+ and CD4+ T-cell priming in draining lymph nodes, effector T-cell trafficking, and tumor cell killing. The right panel summarizes tumor-driven DC dysfunction mediated by immunosuppressive factors within the tumor microenvironment, including VEGF, IL-10, TGF-β, PGE2, adenosine, hypoxia, stromal barriers, and suppressive myeloid cells. These signals impair DC maturation, antigen presentation, IL-12 production, and migration, resulting in weak T-cell priming, T-cell exhaustion or exclusion, reduced responsiveness to immune checkpoint inhibitors, and immune escape. DC abundance, subset composition, functional state, and spatial organization collectively determine whether tumor antigenicity is converted into productive antitumor immunity. cDC1, conventional type 1 dendritic cell; cDC2, conventional type 2 dendritic cell; pDC, plasmacytoid dendritic cell; moDC, monocyte-derived dendritic cell; MHC, major histocompatibility complex; IFN, interferon; VEGF, vascular endothelial growth factor; IL, interleukin; TGF-β, transforming growth factor beta; PGE2, prostaglandin E2; MDSC, myeloid-derived suppressor cell; TAM, tumor-associated macrophage; ICI, immune checkpoint inhibitor.

**Figure 2:**
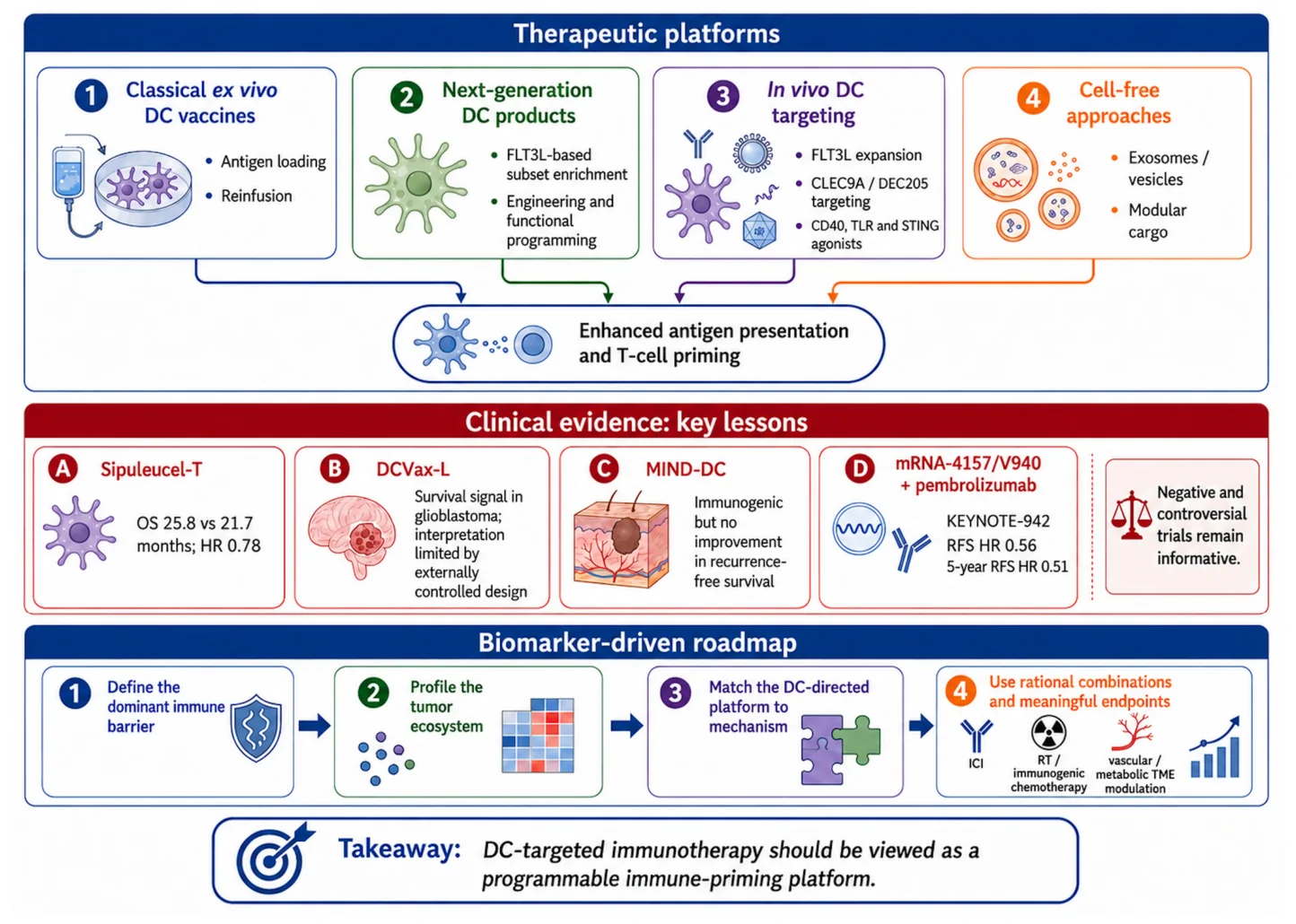
Therapeutic strategies and roadmap for dendritic cell-targeted immunotherapy. The figure summarizes the main dendritic cell (DC)-directed and DC-dependent immunotherapeutic strategies, including classical ex vivo DC vaccines, next-generation DC products, in vivo DC targeting, and cell-free approaches based on exosomes or extracellular vesicles. These platforms converge on enhanced antigen presentation and T-cell priming. Representative clinical examples highlight both validated benefit and unresolved limitations, including sipuleucel-T, DCVax-L, MIND-DC, and individualized neoantigen mRNA vaccination combined with pembrolizumab. The lower panel proposes a biomarker-driven roadmap based on defining the dominant immune barrier, profiling the tumor ecosystem, matching the DC-directed platform to the underlying mechanism, and using rational combinations with clinically meaningful endpoints. OS, overall survival; HR, hazard ratio; RFS, recurrence-free survival; DC, dendritic cell; mRNA, messenger RNA; FLT3L, Fms-like tyrosine kinase 3 ligand; CLEC9A, C-type lectin domain containing 9A; DEC205, dendritic cell receptor 205; TLR, Toll-like receptor; STING, stimulator of interferon genes; ICI, immune checkpoint inhibitor; RT, radiotherapy; TME, tumor microenvironment.

**Table 1:** Comparative summary of DC-directed and DC-dependent immunotherapeutic platforms.

Platform	Main Mechanism	Clinical Maturity	Representative Evidence	Main Limitation
Sipuleucel-T	Autologous APC activation against prostatic acid phosphatase	Approved	IMPACT: OS 25.8 vs. 21.7 months; HR for death 0.78 [9]	OS benefit without clear objective progression endpoint benefit
Tumor lysate-loaded DCs	Broad tumor antigen presentation after ex vivo antigen loading	Phase 2/3 tested	DCVax-L: nGBM OS 19.3 vs. 16.5 months; rGBM OS 13.2 vs. 7.8 months using external controls [10]	External-control design and selection/crossover concerns
Natural DC vaccination	Use of circulating CD1c+ cDCs and pDCs loaded with tumor antigens	Phase 3 tested	MIND-DC: 2-year RFS 36.8% vs. 46.9%; median RFS 12.7 vs. 19.9 months [12]	Immunogenicity did not translate into clinical benefit
FLT3L-based DC expansion/differentiation	Expansion or generation of conventional DC-enriched populations	Preclinical/early translational	FLT3L/GM-CSF-induced DCs enhanced cross-presentation and CD8+ T-cell responses [47]	Expansion alone insufficient without antigen and maturation signals
STING/TLR/CD40 agonists	Innate DC activation, licensing, type I IFN and IL-12 induction	Early clinical/translational	Strong mechanistic rationale and preclinical synergy with ICIs [19,33,34]	Systemic inflammation, delivery constraints, therapeutic window
mRNA neoantigen vaccines	Endogenous APC/DC-dependent antigen expression, processing and presentation	Phase 2b positive	KEYNOTE-942: HR 0.561 primary analysis; 5-year HR 0.51 for recurrence/death [69,70]	Personalized manufacturing, cost, antigen prediction and validation
DC-derived exosomes	Cell-free transfer of MHC-peptide complexes and immunostimulatory cargo	Early phase	Early melanoma and NSCLC trials explored feasibility [60,61]	Vesicle heterogeneity, standardization, biodistribution and potency assays

## Data Availability

No new data were created or analyzed in this study. Data sharing is not applicable to this article.
